# A novel nasal-to-oral airflow pressure ratio as an objective indicator of nasal obstruction

**DOI:** 10.3389/falgy.2026.1807126

**Published:** 2026-04-17

**Authors:** Shiqi Wang, Baoshi Fan, Yuntian Bao, Yusong Dai, Minghui Wang, Xindi Yang, Wendong Liu, Caifeng Xia, Yu Song

**Affiliations:** 1 Department of Otolaryngology-Head and Neck Surgery, Peking University First Hospital, Peking University, Beijing, China; 2 Beijing Institute of Technology, Beijing, China; 3 Peking University Third Hospital, Beijing, China

**Keywords:** nasal airflow pressure, Nasal Obstruction, nasal-to-oral airflow pressure ratio, oral airflow pressure, VAS (analog visual scale)

## Abstract

**Objective:**

To introduce and clinically validate, for the first time, a novel nasal-to-oral airflow pressure ratio as an objective metric for assessing nasal obstruction. This ratio is designed to capture compensatory breathing patterns and to improve correlation with subjective symptoms compared with existing assessment methods.

**Methods:**

A total of 108 patients with self-reported nasal obstruction and 26 healthy controls were enrolled. A custom-designed face mask equipped with pressure sensors recorded nasal airflow pressure during quiet breathing and oral airflow pressure during forced oral breathing. The nasal-to-oral airflow pressure ratio was calculated and correlated with visual analogue scale (VAS) scores for nasal obstruction using Spearman's correlation. Sex-specific subgroup analyses were performed.

**Results:**

The nasal-to-oral airflow pressure ratio was significantly lower in patients with nasal congestion than in controls (0.13 ± 0.10 vs 0.23 ± 0.12; *p* < 0.001). The ratio showed a strong negative correlation with VAS scores (*ρ* = −0.645; *p* < 0.001), outperforming traditional metrics for assessing nasal obstruction. Sex-specific analyses revealed consistent trends in both males and females, with a slightly stronger correlation in female patients.

**Conclusion:**

This study provides the first clinical validation of the nasal-to-oral airflow pressure ratio as a sensitive, practical, and objective indicator of nasal obstruction. By incorporating compensatory oral breathing dynamics, the ratio bridges the gap between subjective sensation and traditional objective measurements, offering a low-cost, easy-to-deploy tool for broad clinical use.

## Introduction

Nasal obstruction is a common complaint in otolaryngology, often causing significant impairment in quality of life and sleep, and affecting up to 30–40% of the general population (1, 2). Understanding the actual nasal ventilation of patients is crucial for diagnosing nasal diseases and assessing treatment effectiveness before and after intervention. Despite its prevalence, accurately assessing nasal obstruction remains a clinical challenge due to the complex interplay between anatomical, physiological, and perceptual factors.

Current methods for assessing the degree of nasal congestion can be divided into subjective assessments and objective measurements. Subjective assessments include the Visual Analogue Scale (VAS) and the Nasal Obstruction Symptom Evaluation (NOSE) scale (3). Objective measurements include rhinomanometry (RMM) (4), acoustic rhinometry (AR) (5), and Nasal Inspiratory Peak Flow (NIPF) (6). Rhinomanometry measures the relationship between airflow and pressure changes in the nasal cavity to calculate nasal resistance. Acoustic rhinometry analyzes the attenuation and phase changes of sound wave reflections within the nasal cavity to calculate cross-sectional area and volume parameters. Nasal inspiratory peak flow refers to the maximum airflow rate achieved during forced inhalation through the nose. Clinically, acoustic rhinometry and rhinomanometry are the most commonly used objective tests. However, studies indicate that both methods have certain limitations.

Several studies have noted a weak correlation between RMM and VAS scores (7). Canakcioglu et al. further confirmed that in patients with normal anatomical structures, nasal congestion can occur due to mucosal congestion, which resistance measurements cannot capture (8, 9). Moreover, rhinomanometry depends on static respiratory instant values, whereas the sensation of nasal congestion relates to airflow patterns during the respiratory cycle. Additionally, acoustic rhinometry involves calculating the nasal cavity's cross-sectional area through sound wave reflections but has low sensitivity in the nasal vestibule area. Ohki et al. pointed out that significant errors occur 1–2 cm into the front of the nasal cavity, which is a key aerodynamics site (10, 11). Also, there is still no clear direct correlation between the nasal cavity's cross-sectional area and subjective nasal congestion perception.

Given this, scholars have recently proposed using nasal inspiratory peak flow to assess the degree of nasal congestion (6). However, due to variations in baseline respiratory flow among different patients, it is challenging to precisely define the degree of nasal congestion using absolute values of nasal inspiratory peak flow. Additionally, previous studies have shown that the trigeminal nerve endings in the nasopharynx perceive changes in airflow temperature/pressure, and when congested, the brainstem respiratory center inhibits nasal breathing reflexes while activating oral breathing mode. This results in a compensatory increase in oral breathing depth during nasal congestion (12, 13). Considering these factors, we believe that simultaneously measuring nasal and oral breathing flow pressure, as well as their ratio, could serve as a more valuable objective indicator of nasal congestion. In this study, we examine the correlation between these measurements and VAS scores to determine their testing effectiveness.

## Materials and methods

### Study design and participants

This prospective observational study was conducted in the Department of Otorhinolaryngology at Peking University First Hospital between September 2024 and March 2025. Participants were recruited through outpatient clinics using flyers and online platforms. All participants underwent a comprehensive otorhinolaryngological physical examination by an experienced otorhinolaryngologist, including anterior rhinoscopy and etc. Inclusion criteria were: (1) age between 18 and 65 years; (2) no history of severe cardiopulmonary or neuropsychiatric disorders; and (3) ability to cooperate with breathing instructions. Exclusion criteria included: (1) presence of large obstructive lesions in the oral cavity, oropharynx, or hypopharynx; (2) diagnosed obstructive sleep apnoea-hypopnoea syndrome (OSAHS); (3) acute upper respiratory tract infection or recent lower airway disease.

A total of 136 subjects with subjective nasal obstruction meeting the inclusion criteria and 43 healthy control subjects were initially enrolled. All eligible participants underwent oronasal airflow pressure detection and VAS scores. After excluding non-standard airflow data due to coughing, speaking, air leakage, or obvious non-cooperation during measurement (see Supplementary Material for examples of excluded curves), 108 subjects with subjective nasal obstruction and 26 normal controls remained for final analysis.

All participants provided written informed consent, and the study protocol was approved by the institutional ethics committee (Ethics Review Number: 2025R0165-0001).

### Airflow measurement system

**Figure 1:**
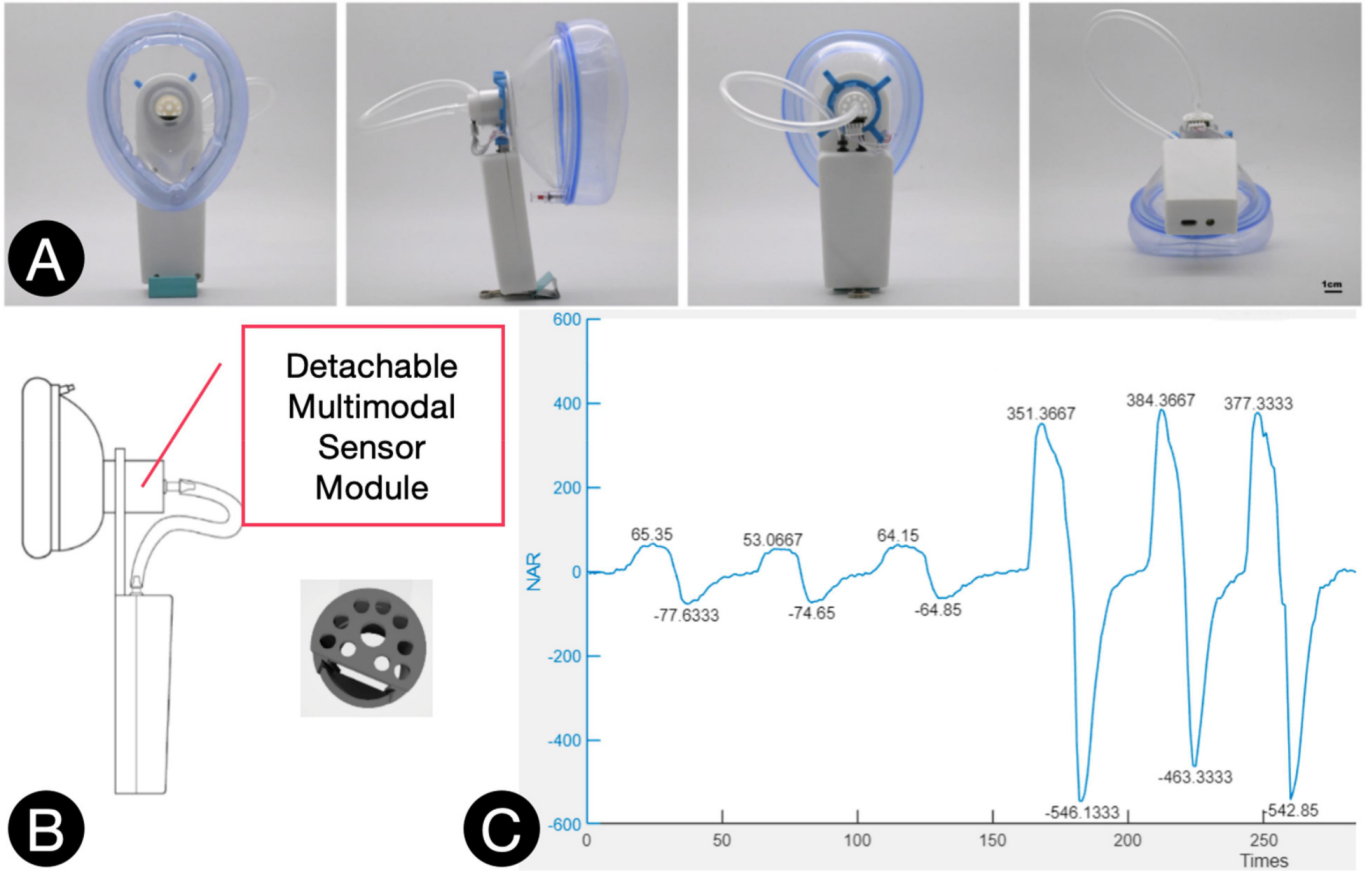
**Airflow measurement system. (A)**: Physical Diagram of the Nasal-Oral Airflow Pressure Collection Mask. **(B)**: Schematic Diagram of the Mask. **(C)**: Representative Curve of Airflow Pressure-Time Curve.

A custom-designed face mask equipped with a differential pressure sensor was developed by the authors to measure nasal and oral airflow pressure (Figure 1) (Patent Number Granted: ZL202420378042.8). The system incorporates a Sensirion SDP610 differential pressure sensor (measurement range: −500 to +500 Pa; accuracy: 0.1 Pa; response time: 4.6 ms; maximum overpressure: 25 kPa). Pressure data were transmitted wirelessly to a computer and converted into pressure–time curves using dedicated software.

Flow rate (Q) was derived from the measured differential pressure (ΔP) based on the Bernoulli equation and the continuity equation, assuming incompressible, steady, and inviscid flow. The relationship is given by $Q=K\sqrt{\Delta P}$, where *K* is a calibration constant determined experimentally. Calibration was performed using a BY-DBT-1 calibration syringe (nominal volume: 3000 mL ± 0.4%; dead space: 190 mL ± 10 mL; accuracy compliant with JJF 1213-2008). The syringe was connected to the mask to generate known standard flow rates, and the corresponding sensor outputs were recorded. Linear regression analysis was applied to the calibration data points ($x_{i}=\sqrt{\Delta P_{i}},Q_{i}$) to estimate *K* as

$$K=\frac{\sum_{i=1}^{n}\;x_{i}Q_{i}}{\sum_{i=1}^{n}\;x_{i}^{2}}$$

Calibration was accepted only if the error was within ±3%; otherwise, the procedure was repeated. This calibration method ensures accurate conversion of the measured pressure signals into airflow values.

### Measurement procedure

Participants were instructed to remove any facial obstructions (e.g., glasses) and wear the mask with the air valves properly adjusted to ensure airtight contact. After brief instruction, each participant underwent the following breathing sequence:

Nasal airflow test: Participants were instructed to “please close your mouth, and breathe quietly through your nose—take a deep, calm breath in, and then breathe out calmly.” Three consecutive cycles of quiet nasal breathing were recorded with the mouth closed.
Oral airflow test: Participants were instructed to “please pinch your nose, and take a deep, quick breath in through your mouth, then breathe out forcefully and quickly, as if you were blowing out birthday candles.” To ensure proper technique, each participant performed three practice trials before data collection. Following practice, three consecutive cycles of forceful oral breathing were recorded with the nose pinched occluded. To ensure measurement reproducibility, the variability of oral expiratory peak flow pressure across the three recorded trials was assessed: if the difference between the highest and lowest values exceeded 25% of the mean, the measurement was repeated.

### Subjective assessment

All participants completed a VAS score for nasal obstruction at the time of testing, ranging from 0 (no obstruction) to 10 (complete obstruction). The VAS score was used as the subjective comparator for evaluating correlation with airflow pressure measurements.

#### Statistical analysis

Data were analysed using SPSS version 27.0 (IBM Corp., Armonk, NY, USA). The Kolmogorov–Smirnov test was used to assess normality of distribution. Between-group comparisons were made using the Mann–Whitney U test for non-normally distributed variables. Correlations between airflow pressure metrics and VAS scores were assessed using Spearman's rank correlation coefficient. To further adjust for potential confounding factors, multivariable linear regression analysis was performed with VAS score as the dependent variable and the nasal-to-oral airflow pressure ratio, sex, and age as independent variables. This analysis aimed to evaluate whether the nasal-to-oral ratio independently predicted subjective nasal obstruction after controlling for sex and age. Subgroup analyses were performed by sex to explore gender-related airflow pressure differences. A p-value < 0.05 was considered statistically significant.

## Results

### Participant characteristics

A total of 134 participants were included in the final analysis: 108 patients with self-reported nasal obstruction (obstruction group) and 26 healthy controls (control group). The mean age was comparable between the two groups (37.2 ± 13.5 years vs. 38.0 ± 10.8 years, *p* = 0.469). However, the sex distribution differed significantly, with a higher proportion of females in the control group (76.9% vs. 46.3%, *p* = 0.005). Detailed demographic characteristics are shown in Table 1.

**Table 1:** Participant demographics.

Characteristic	Obstruction Group (*n* = 108)	Control Group (*n* = 26)	*p* value
**Age (years), mean ± SD**	37.2 ± 13.5	38.0 ± 10.8	0.469
**Male, n (%)**	58 (53.7%)	6 (23.1%)	0.005
**Female, n (%)**	50 (46.3%)	20 (76.9%)	

### Airflow measurements

The mean peak nasal inspiratory airflow pressure was significantly lower in the obstruction group compared to controls (31.4 ± 23.3 vs. 42.0 ± 23.5; *p* = 0.015). In contrast, the mean peak oral expiratory airflow pressure was significantly higher in the obstruction group (292.0 ± 184.4 vs. 216.2 ± 152.8; *p* = 0.038), suggesting a compensatory increase in oral breathing.

Importantly, the nasal-to-oral airflow pressure ratio was markedly reduced in the obstruction group compared to controls (0.13 ± 0.10 vs. 0.23 ± 0.12; *p* < 0.001), indicating a shift toward oral breathing in patients with nasal airflow limitation (Table 2).

**Table 2:** Airflow pressure measurements in obstruction and control groups.

Variable	Obstruction Group (*n* = 108)	Control Group (*n* = 26)	*p* value
**Nasal inspiratory peak flow pressure (mean ± SD)**	31.4 ± 23.3	42.0 ± 23.5	0.015
**Oral expiratory peak flow pressure (mean ± SD)**	292.0 ± 184.4	216.2 ± 152.8	0.038
**Nasal-to-oral airflow pressure ratio**	0.13 ± 0.10	0.23 ± 0.12	<0.001

### Correlation with subjective nasal obstruction

Spearman’s correlation analysis demonstrated a strong negative correlation between the nasal-to-oral airflow pressure ratio and VAS scores (*ρ* = −0.645, *p* < 0.001), indicating that lower ratios were associated with greater subjective nasal obstruction.

Multivariable linear regression analysis, adjusting for sex and age, showed that the nasal-to-oral airflow pressure ratio was a significant independent predictor of VAS score (B = −12.38, *β* = −0.537, *P* < 0.001), explaining 29.7% of the variance (adjusted R^2^ = 0.297). Neither sex (*P* = 0.529) nor age (*P* = 0.138) was significantly associated with VAS scores. These findings confirm that the nasal-to-oral ratio correlates with subjective nasal obstruction independently of demographic factors, supporting its robustness as an objective indicator.

**Figure 2:**
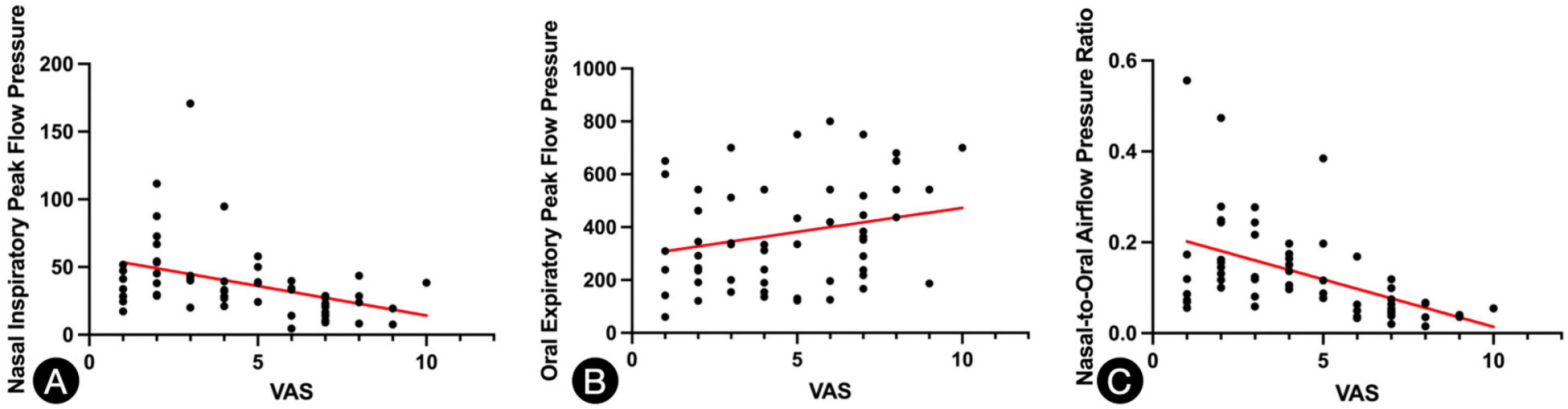
**Spearman's correlation analysis. (A)**: Fitted Linear Regression Curve of Nasal Inspiratory Peak Flow Pressure vs. VAS score. **(B)**: Fitted Linear Regression Curve of Oral Expiratory Peak Flow Pressure vs. VAS score. **(C)**: Nasal-to-Oral Airflow Pressure Ratio vs. VAS score.

Additionally, nasal inspiratory airflow pressure alone was moderately negatively correlated with VAS scores (r = –0.493, *p* < 0.001), while oral airflow pressure was weakly and non-significantly positively correlated with VAS (r = 0.173, *p* = 0.074). These results are summarised in Table 3 and visualised in Figure 2.

**Table 3:** Correlation between airflow pressure metrics and VAS scores (*n* = 108).

Metric	Spearman's *ρ*	*p* value
**Nasal inspiratory peak flow pressure vs. VAS**	–0.493	<0.001
**Oral expiratory peak flow pressure vs. VAS**	0.173	0.074
**Nasal-to-oral airflow pressure ratio vs. VAS**	−0.645	<0.001
**Nasal vs. oral airflow pressure (internal correlation)**	0.437	<0.001

### Sex-specific subgroup analysis

Among participants in the obstruction group, males had significantly higher nasal (38.2 ± 27.2 vs. 23.5 ± 14.4; *p* < 0.001) and oral (372.9 ± 192.5 vs. 198.1 ± 114.2; *p* < 0.001) airflow pressure values compared to females, consistent with known anatomical and physiological differences.

However, there was no significant difference in the nasal-to-oral airflow pressure ratio between sexes (male: 0.13 ± 0.11; female: 0.14 ± 0.09; *p* = 0.169), nor in VAS scores (male: 4.50 ± 2.52; female: 4.56 ± 1.90; *p* = 0.751).

Interestingly, the correlation between the nasal-to-oral ratio and VAS was stronger in females (r = –0.726) than in males (r = –0.610), suggesting a slightly more consistent relationship in women (Table 4).

**Table 4:** Sex-Specific airflow pressure analysis Among obstruction group.

Variable	Male (*n* = 58)	Female (*n* = 50)	*p* value
**Nasal inspiratory peak flow pressure**	38.17 ± 27.17	23.48 ± 14.40	<0.001
**Oral expiratory peak flow pressure**	372.89 ± 192.51	198.05 ± 114.23	<0.001
**Nasal-to-oral airflow pressure ratio**	0.13 ± 0.11	0.14 ± 0.09	0.169
**VAS score**	4.50 ± 2.52	4.56 ± 1.90	0.751
**ρ: Ratio vs. VAS**	–0.610	–0.726	<0.001

## Discussion

This study proposes a novel physiological index—the nasal-/oral airflow pressure ratio—to objectively quantify nasal obstruction. By simultaneously measuring nasal and oral airflow pressure during controlled respiratory maneuvers, this ratio reflects not only the degree of nasal patency but also the extent of compensatory oral breathing. Our findings suggest that this ratio demonstrates stronger correlation with patient-reported nasal obstruction (VAS scores) than either nasal airflow pressure or oral airflow pressure alone, and it may serve as a useful adjunct to traditional objective assessments.

### Comparison with conventional objective measures

Multiple prior studies have emphasized the modest correlation between objective nasal patency tests and subjective symptoms. For example, Ottaviano et al. reported a Spearman's correlation coefficient of –0.45 between PNIF and VAS in a large cohort (*n* > 1000), indicating limited but clinically relevant alignment between the two measures (14). Similarly, studies on rhinomanometry and acoustic rhinometry have shown variable and often weak associations with subjective nasal obstruction scores (7).

In our study, the nasal-oral airflow pressure ratio yielded a stronger negative correlation with VAS scores (*ρ* = −0.645, *p* < 0.001) than nasal airflow alone (*ρ* = –0.493) or oral airflow alone (non-significant). These findings support the hypothesis that the ratio captures not only absolute nasal airflow pressure, but also a compensatory respiratory response that contributes to patients’ overall perception of obstruction. This dual-pathway measurement may offer better clinical sensitivity, particularly in patients whose nasal airflow is borderline but who still report significant obstruction symptoms.

### Physiological rationale

The rationale for this method is based on established neurophysiological responses to nasal resistance. Studies have shown that the trigeminal afferents in the nasal mucosa respond to changes in temperature and airflow dynamics and contribute to central modulation of respiratory drive (15). When nasal airflow is reduced, oral breathing may be reflexively enhanced to maintain adequate ventilation (16). Our measurement protocol—capturing nasal inspiratory flow pressure during quiet breathing and oral flow pressure during voluntary deep breaths—allows estimation of this compensatory adjustment. The resulting ratio reflects the functional shift in airflow distribution between the two routes.

Moreover, the use of a ratio mitigates interindividual variability in baseline pulmonary function, body habitus, and inspiratory effort. Unlike PNIF, which is effort-dependent and significantly influenced by sex, height, and lung volume, a ratio inherently normalizes individual variability (14, 17). This is supported by our data showing no significant sex difference in the nasal-oral airflow pressure ratio (*p* = 0.169), despite marked differences in absolute airflow pressure values between male and female participants.

However, we acknowledge that this ratio is not determined solely by nasal patency. Other structures of the upper airway, particularly the functional status of the velopharynx, may also influence airflow distribution. During forced expiration, the contraction efficiency of muscles such as the levator veli palatini (i.e., velopharyngeal closure efficiency) directly affects oral airflow expulsion and nasal airflow leakage. Although the influence of velopharyngeal function may be minimized under the specific measurement conditions of quiet nasal breathing and forced oral breathing, future studies incorporating nasopharyngoscopy or advanced aerodynamic modeling would help to more precisely delineate the contributions of nasal and velopharyngeal factors to this ratio.

### Clinical implications

The nasal-oral airflow pressure ratio may hold clinical utility in several scenarios: As a screening tool for nasal obstruction, particularly when rhinomanometry or acoustic rhinometry is unavailable; In pre- and post-treatment comparisons, to evaluate improvement following medical or surgical intervention; In patients with functional nasal obstruction, where anatomical imaging and airflow resistance values are inconclusive; In gender-diverse populations, where absolute flow thresholds may lack consistent clinical meaning.

The measurement device used in this study is compact, relatively low-cost, and does not require advanced calibration or specialized technical expertise, making it feasible for routine clinical use, including in primary care or community settings.

### Limitations

Several limitations of this study should be acknowledged. First, although we confirmed strong cross-sectional correlation with subjective symptoms, this study did not assess responsiveness to treatment. Therefore, it remains to be established whether changes in the nasal-oral ratio correspond reliably with clinical improvement after decongestants, corticosteroids, or surgery.

Second, the methodology requires patient cooperation and controlled breathing efforts. While this was feasible in our adult cohort, future studies should determine whether similar reliability can be obtained in pediatric or geriatric populations. Third, this study did not compare the nasal-oral airflow pressure ratio directly with established methods such as rhinomanometry or acoustic rhinometry in the same patient group. Such head-to-head comparison would provide more robust evidence of its relative diagnostic value.

Finally, due to the recruitment strategy, the proportion of female participants was higher in the healthy control group. Although subgroup analysis indicated that sex did not significantly influence the nasal-oral ratio, future studies with balanced gender composition are warranted to validate these results.

## Conclusion

The nasal-to-oral airflow pressure ratio is a promising objective indicator for nasal obstruction. Compared to traditional objective methods, it demonstrates a stronger correlation with subjective symptom scores and incorporates compensatory oral breathing dynamics, enhancing its clinical relevance and diagnostic value for nasal obstruction. This method may offer advantages over traditional techniques by incorporating compensatory oral breathing into the evaluation framework.

## Data Availability

The original contributions presented in the study are included in the article/Supplementary material, further inquiries can be directed to the corresponding authors.
