# Supplementary material for: A novel nasal-to-oral airflow pressure ratio as an objective indicator of nasal obstruction
Source: Front Allergy. 2026 Apr 17;7:1807126. doi: 10.3389/falgy.2026.1807126 (PMC13132843; doi:10.3389/falgy.2026.1807126)
Supplement: Supplementary file 1 [file Supplementaryfile1.pdf]

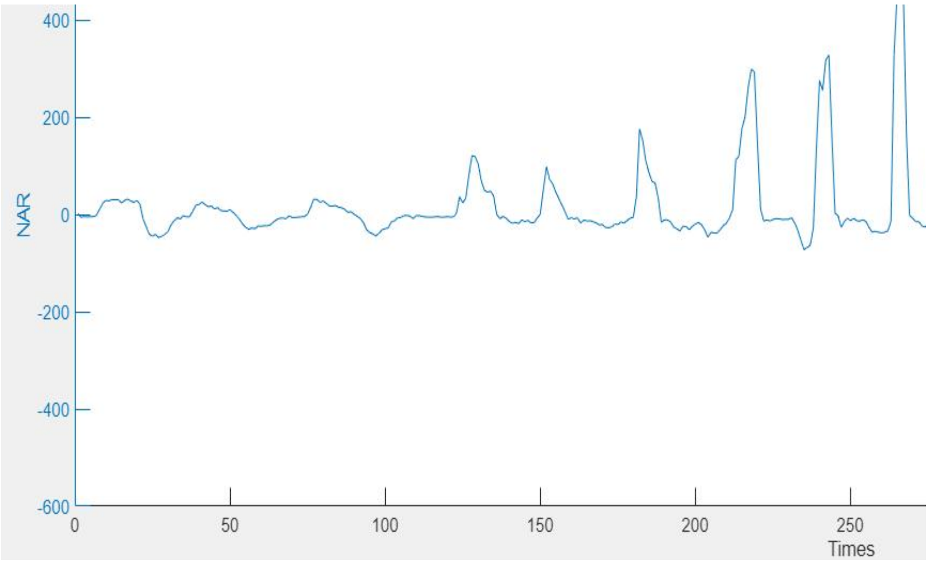

Abnormal Curve 1: Among the first three respiratory curves, only the first one is satisfactory, while the latter two show poor stability.

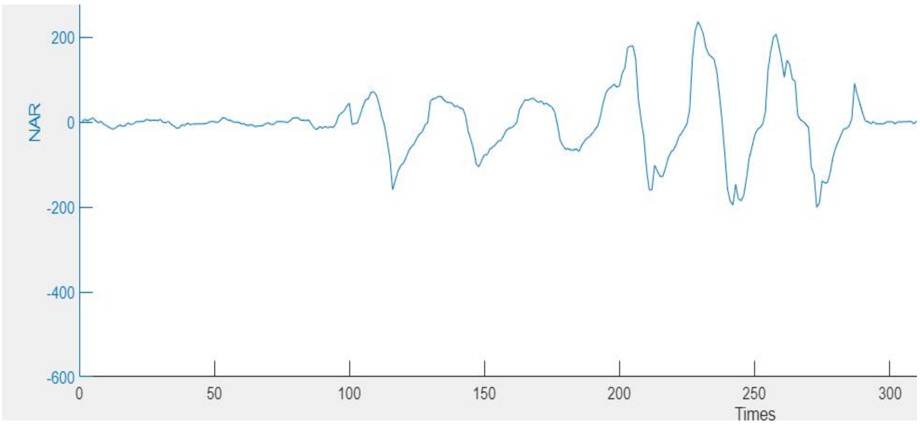

Abnormal Curve 2: Among the first three respiratory curves, only the third one is satisfactory, while the first respiratory curve shows obvious signs of interruption.

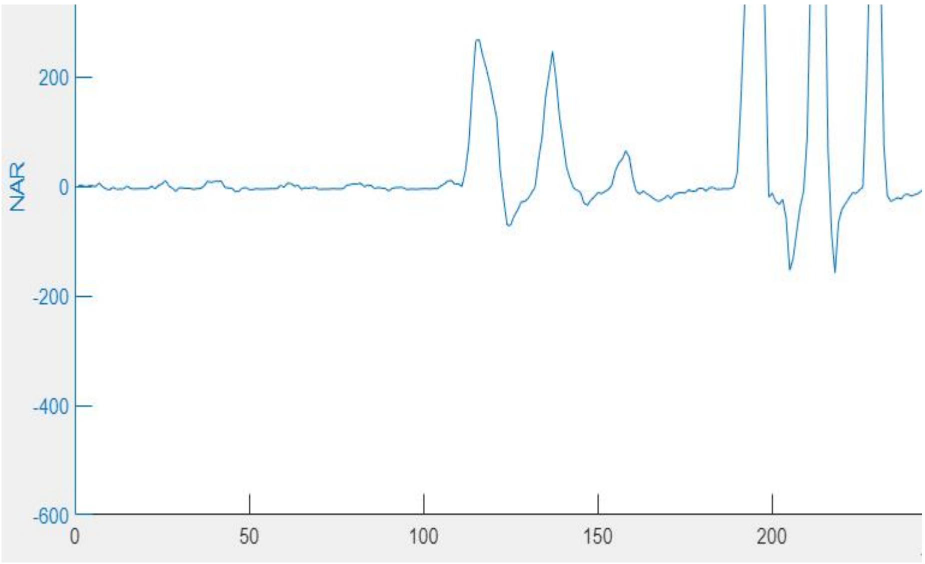

Abnormal Curve 3: The peak value of the third respiratory curve is significantly lower than those of the first two.
